# Global-scale parameters for ecological models

**DOI:** 10.1038/s41597-022-01904-3

**Published:** 2023-01-04

**Authors:** Gianpaolo Coro, Pasquale Bove, Kathleen Kesner-Reyes

**Affiliations:** 1 Institute of Information Science and Technologies, Italian National Research Council, Pisa, 56124 Italy; 2 Quantitative Aquatics, Inc., Los Baños, 4031 Laguna, Philippines

**Keywords:** Marine biology, Physical oceanography, Ecological modelling

## Abstract

This paper presents a collection of environmental, geophysical, and other marine-related data for marine ecological models and ecological-niche models. It consists of 2132 raster data for 58 distinct parameters at regional and global scales in the ESRI-GRID ASCII format. Most data originally belonged to open data owned by the authors of this article but residing on heterogeneous repositories with different formats and resolutions. Other data were specifically created for the present publication. The collection includes 565 data with global scale range; 154 at 0.5° resolution and 411 at 0.1° resolution; 196 data with annual temporal aggregation over ~10 key years between 1950 and 2100; 369 data with monthly aggregation at 0.1° resolution from January 2017 to ~May 2021 continuously. Data were also cut out on 8 European marine regions. The collection also includes forecasts for different future scenarios such as the Representative Concentration Pathways 2.6 (63 data), 4.5 (162 data), and 8.5 (162 data), and the A2 scenario of the Intergovernmental Panel on Climate Change (180 data).

## Abstract (summary)

**Table:** 

Measurement(s)	habitat similarity • Time Series Analysis • Principal Component
Technology Type(s)	Habitat Representativeness Score • Cross-Correlation • Principal Component Analysis
Factor Type(s)	Sea-bottom and sea-surface dissolved oxygen, salinity, and temperature • Sea net primary production • Sea ice concentration • Average, minimum, maximum sea depth • Average, minimum, maximum elevation • Distance of a square marine area from land and its fraction covered by water • Indication of the belonging Large Marine Ecosystem (LME), Exclusive Economic Zone (EEZ), Marine Ecoregions of the World (MEOW), Major Ocean Basins, and Marine Protected Area (MPA) • Number of islands • Water area that lies within the shelf, slope, and abyssal zones • Tidal range extension • Coral density • Estuary and seamount presence • Carbon dioxide flux at soil • Air surface temperature • Precipitation • Difference between air surface temperature and sea surface temperature • World population density • Sediment thickness • Atmospheric concentration of methane and nitrous oxide • Earth heat flow • Distance from crust plates • Earthquake density, depth, magnitude • Groundwater resources
Sample Characteristic - Environment	climate system
Sample Characteristic - Location	Oceans and Seas • Adriatic Sea • Aegean Sea • Baltic Sea • Bay of Biscay • Black Sea • Mediterranean Sea, Eastern Basin • North Sea • Mediterranean Sea, Western Basin

## Background & Summary

The Good Environmental Status of European Seas (GES)^1^ is the European goal of reaching the sustainably of stock and environment exploitation and no loss of biodiversity and ecosystem services. It is the primary goal of several European strategic frameworks such as the Marine Strategy Framework Directive (MSFD), the Maritime Spatial Planning Directive (MSP), the Green Deal and Blue Growth strategies, and the EU Biodiversity Strategy for 2030^2–7^. This goal is challenging in the current context of increasing energy, food demand, and climate change. Scientific approaches that address GES require processing marine data of ecosystems to assess ecosystem services, biodiversity, and stock status. They also require multi-disciplinary modelling approaches to extract valuable knowledge from the data^6^. Recently, international projects such as EcoScope^8^, have been fostering the shift from traditional “vertical” modelling approaches - focussing on one species, stock, or ecosystem service independently of the other - to “horizontal” approaches, which combine multi-species, environmental, and social dynamics^9,10^. However, these approaches require huge amounts of high-quality data to produce meaningful knowledge^11,12^. In particular, environmental, geophysical, world-population, and marine-region data are crucial to model species habitats^13,14^, understand the response and resilience of marine areas to climate change^15–17^, assess stock status and fisheries pressure on stocks^18–20^, and build ecosystem models^21–24^.

This paper describes an extensive data collection of harmonised and standardised global-scale parameters, with associated long-term forecasts under different greenhouse gas emission and societal development scenarios. The collection aims at supporting ecological, ecosystem, and ecological-niche models within horizontal approaches to marine resource management.

**Fig. 1:**
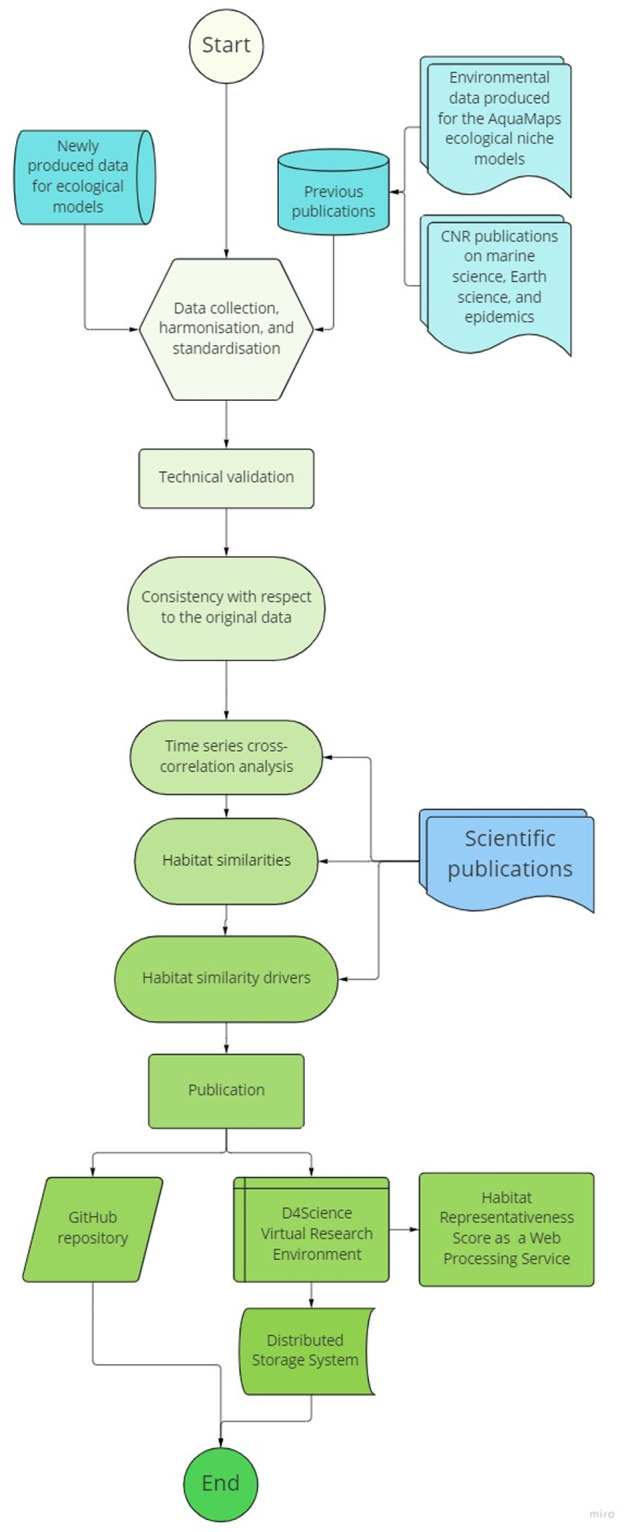
Conceptual flowchart of our data harmonisation, validation, and publication workflow.

Figure 1 summarises our workflow. We harmonised and standardised geospatial data from our own heterogeneous resources and publications that had newly produced or re-processed these data. Some data were previously available in custom formats (e.g., CSV or text files), which meant they were not as accessible as they could be. Additionally, we specifically produced other data to complement the collection. The primary sources involved were (i) environmental data produced for the AquaMaps ecological niche models, (ii) data from the Italian National Research Council (CNR) studies on marine science, Earth science, and epidemics that re-processed or newly produced open-access data based on other sources, and (iii) data produced by the Quantitative Aquatics (Q-quatics) non-governmental organisation for ecosystem and ecological models.

The complete list of environmental data with their primary and secondary sources is reported in Tables 1–4, grouped by resolution and parameter type. Data harmonisation consisted of correcting errors and aligning the data to the same coordinate grids, with either 0.1° or 0.5° resolutions. The format of the published data is ESRI-GRID ASCII. All data have a global-scale range but are also cut out on 8 European marine areas of particular economic or ecosystem importance (*focus regions*), identified by the EcoScope European Project community of practice^8^. This specialisation aims to make the collection highly valuable for European and global-scale ecological niche models, ecological models, ecosystem models, environmental similarity analyses, and climate change studies, as also documented in the rest of the paper.

**Table 1:** Data at 0.1° resolution at the global scale available in our repository, with indication of the related primary and secondary sources. The asterisks (*) indicate the data that were specifically produced for this article.

0.1° resolution - global scale data						
Parameter name	Description	Unit of measurement	Years or temporal aggregation	Original File Format	Primary source	Secondary sources
Sea-bottom dissolved oxygen*	Average dissolved molecular oxygen at sea bottom	mmol *m*^−3^	2017,2018,2019,2020, monthly from Jan 2017 to May 2021	ESRI-GRID (ASC)	Produced by Q-quatics for this publication	Bio-ORACLE^107,108^ and CMEMS^45^
Sea-bottom salinity*	Average sea bottom salinity	PSS	2017,2018,2019,2020, monthly from Jan 2017 to Mar 2021	ESRI-GRID (ASC)	Produced by Q-quatics for this publication	Bio-ORACLE^107,108^ and CMEMS^45^
Sea-bottom temperature*	Average temperature at sea bottom	°C	2016,2017,2018,2019,2020	ESRI-GRID (ASC)	Produced by Q-quatics for this publication	Bio-ORACLE^107,108^ and CMEMS^45^
Sea Net Primary Production*	Average sea surface primary production in a cell, re-processed from Bio-ORACLE data	*mgC m*^−3^ *day*^−1^	2017,2018,2019,2020, monthly from Jan 2017 to May 2021	ESRI-GRID (ASC)	Produced by Q-quatics for this publication	Bio-ORACLE^107,108^ and CMEMS^45^
Sea Ice Concentration*	Average sea ice concentration ratio per cell	0–1 fraction	2016,2017,2018,2019,2020, monthly from Jan 2017 to May 2021	ESRI-GRID (ASC)	Produced by Q-quatics for this publication	Bio-ORACLE^107,108^ and CMEMS^45^
Sea-surface Salinity*	Average sea surface salinity	PSS	2016,2017,2018,2019,2020, monthly from Jan 2017 to May 2021	ESRI-GRID (ASC)	Produced by Q-quatics for this publication	Bio-ORACLE^107,108^ and CMEMS^45^
Sea-surface Temperature*	Average temperature at sea surface	°C	2016,2017,2018,2019,2020, monthly from Jan 2017 to May 2021	ESRI-GRID (ASC)	Produced by Q-quatics for this publication	Bio-ORACLE^107,108^ and CMEMS^45^
Carbon dioxide flux at soil surface	Average monthly carbon dioxide flux at the soil surface	gC *m*^−2^ *day*^−1^	1979–2013	ESRI-GRID (ASC)	Coro & Bove (2022)^109^	Copernicus Atmosphere Monitoring Service data^110^
Mean Air Surface Temperature	Average annual surface air temperature between 2000 and 2005	K	2000–2005	ESRI-GRID (ASC)	Coro & Bove (2022)^109^	EnviDat^111^
Mean Precipitation	Average annual precipitation between 2000 and 2005	kg *m*^−2^ *s*^−1^	2000–2005	ESRI-GRID (ASC)	Coro & Bove (2022)^109^	EnviDat^111^

**Table 2:** Data of marine parameters at 0.5° resolution at the global scale available in our repository, with indication of the related primary and secondary sources.

Marine parameters - 0.5° resolution global-scale data							
Parameter name	Description	Unit of measurement	Years or temporal aggregation	Climatic forecast scenarios	Original File Format	Primary source	Secondary sources
Sea-bottom dissolved oxygen	Average dissolved molecular oxygen at sea bottom	mmol *m*^−3^	2019, 2050, 2100	RCP 2.6, RCP 4.5, RCP 8.5	ESRI-GRID (ASC)	AquaMaps HCAF V7^112^	Bio-ORACLE^107,108^
Sea-bottom salinity	Average sea bottom salinity	PSS	2019, 2050, 2100	RCP 2.6, RCP 4.5, RCP 8.5	ESRI-GRID (ASC)	AquaMaps HCAF V7^112^	Bio-ORACLE^107,108^
Sea-bottom temperature	Average temperature at sea bottom	°C	2019, 2050, 2100	RCP 2.6, RCP 4.5, RCP 8.5	ESRI-GRID (ASC)	AquaMaps HCAF V7^112^	Bio-ORACLE^107,108^
Sea Net Primary Production	Average sea surface primary production in a cell	mgC *m*^−3^ *day*^−1^	2019, 2050, 2100	RCP 2.6, RCP 4.5, RCP 8.5	ESRI-GRID (ASC)	AquaMaps HCAF V7^112^	Bio-ORACLE^107,108^
Sea Ice Concentration	Average sea ice concentration ratio per cell	0–1 fraction	2019, 2050, 2100	RCP 2.6, RCP 4.5, RCP 8.5	ESRI-GRID (ASC)	AquaMaps HCAF V7^112^	Bio-ORACLE^107,108^
Sea-surface salinity	Average sea surface salinity	PSS	2019, 2050, 2100	RCP 2.6, RCP 4.5, RCP 8.5	ESRI-GRID (ASC)	AquaMaps HCAF V7^112^	Bio-ORACLE^107,108^
Sea-surface temperature	Average temperature at sea surface	°C	2019, 2050, 2100	RCP 2.6, RCP 4.5, RCP 8.5	ESRI-GRID (ASC)	AquaMaps HCAF V7^112^	Bio-ORACLE^107,108^
Sea-bottom salinity - AquaMaps2016	Average sea bottom salinity	PSS	1950,1999,2016,2050,2100	IPCC SRES A2	NetCDF	Coro *et al*.^17^	AquaMaps HCAF V6^113^
Sea-bottom temperature - AquaMaps2016	Average temperature at sea bottom	°C	1950,1999,2016,2050,2100	IPCC SRES A2	NetCDF	Coro *et al*.^17^	AquaMaps HCAF V6^113^
Net Primary Production - AquaMaps2016	Annual sea surface primary production in a cell	mgC *m*^−2^ *day*^−1^	1950,1999,2016,2050,2100	IPCC SRES A2	NetCDF	Coro *et al*.^17^	AquaMaps HCAF V6^113^
Sea Ice Concentration - AquaMaps2016	Average sea ice concentration ratio per cell	0–1 fraction	1950,1999,2016,2050,2100	IPCC SRES A2	NetCDF	Coro *et al*.^17^	AquaMaps HCAF V6^113^
Sea-surface salinity - AquaMaps2016	Average sea surface salinity	PSS	1950,1999,2016,2050,2100	IPCC SRES A2	NetCDF	Coro *et al*.^17^	AquaMaps HCAF V6^113^
Sea-surface temperature - AquaMaps2016	Average temperature at sea surface	°C	1950,1999,2016,2050,2100	IPCC SRES A2	NetCDF	Coro *et al*.^17^	AquaMaps HCAF V6^113^

**Table 3:** Data of geophysical parameters at 0.5° resolution at the global scale available in our repository, with indication of the related primary and secondary sources.

Geophysical parameters - 0.5° resolution global-scale data							
Parameter name	Description	Unit of measurement	Years or temporal aggregation	Climatic forecast scenarios	Original File Format	Primary source	Secondary sources
Air Surface Temperature	Average air temperature at the Earth surface	K	1950,1999,2016,2050,2100	RCP 4.5, RCP 8.5	NetCDF	Coro *et al*.^17^	NASA-NEX model ensemble^114^
Precipitation	Average precipitation	kg *m*^−2^ *s*^−1^	1950,1999,2016,2050,2100	RCP 4.5, RCP 8.5	NetCDF	Coro *et al*.^17^	NASA-NEX model ensemble^114^
Difference between Air Surface Temperature and Sea Surface Temperature	Difference between average air temperature at the Earth surface and average temperature at the sea surface	°C	1950,1999,2016,2050,2100	RCP 4.5, RCP 8.5	NetCDF	Coro *et al*.^17^	NASA-NEX model ensemble^114^, AquaMaps HCAF V6^113^
Gas concentration of methane (CH4)	Column-mean atmospheric dry mole fraction of methane (CH4)	10^−9^ *mol*^−1^	2019		ASCII Gridded (XYZ)	Coro (2020)^115^	Copernicus Atmosphere Monitoring Service data^116^
Gas concentration of nitrous oxide (N2O)	Column-mean atmospheric dry mole fraction of nitrous oxide (N2O)	10^−9^ *mol*^−1^	2019		ASCII Gridded (XYZ)	Coro (2020)^115^	Copernicus Atmosphere Monitoring Service data^116^
Minimum depth	Minimum bathymetry	m	2019		ESRI-GRID (ASC)	AquaMaps HCAF V7^112^	ETOPO2^117^
Maximum depth	Maximum bathymetry	m	2019		ESRI-GRID (ASC)	AquaMaps HCAF V7^112^	ETOPO2^117^
Mean depth	Average bathymetry	m	2019		ESRI-GRID (ASC)	AquaMaps HCAF V7^112^	ETOPO2^117^
Elevation Min	Minimum elevation above sea level	m	2019		CSV	AquaMaps HCAF V7^112^	ETOPO2^117^
Elevation Max	Maximum elevation above sea level	m	2019		CSV	AquaMaps HCAF V7^112^	ETOPO2^117^
Elevation Mean	Average elevation above sea level	m	2019		CSV	AquaMaps HCAF V7^112^	ETOPO2^117^
Elevation SD	Standard deviation of elevation above sea level	m	2019		CSV	AquaMaps HCAF V7^112^	ETOPO2^117^
Elevation/Depth	A global dataset of elevation and depth	m	2019		NetCDF	Coro & Trumpy (2020)^118^	ETOPO2^117^
Distance from land	Distance of water cells to the nearest coastal cell	km	2019		ESRI-GRID (ASC)	AquaMaps HCAF V7^112^	
Ocean Area	The area in the cell that is normally covered by sea water or permanent ice	*km*^2^	2019		CSV	AquaMaps HCAF V7^112^	
Ocean Basin	Major ocean basins of the world (codes)	—	2019		CSV	AquaMaps HCAF V7^112^	
Islands No	Number of coastal or oceanic islands contained in the cell	—	2019		CSV	AquaMaps HCAF V7^112^	World Vector Shoreline database^119^
Area 0_20	Water area per cell from 0 to 20 m depth	*km*^2^	2019		CSV	AquaMaps HCAF V7^112^	Tozer *et al*.^120^
Area 20_40	Water area per cell from 20 to 40 m depth	*km*^2^	2019		CSV	AquaMaps HCAF V7^112^	Tozer *et al*.^120^
Area 40_60	Water area per cell from 40 to 60 m depth	*km*^2^	2019		CSV	AquaMaps HCAF V7^112^	Tozer *et al*.^120^
Area 60_80	Water area per cell from 60 to 80 m depth	*km*^2^	2019		CSV	AquaMaps HCAF V7^112^	Tozer *et al*.^120^
Area 80_100	Water area per cell from 80 to 100 m depth	*km*^2^	2019		CSV	AquaMaps HCAF V7^112^	Tozer *et al*.^120^
Area Below 100	Water area per cell below 100 m depth	*km*^2^	2019		CSV	AquaMaps HCAF V7^112^	Tozer *et al*.^120^
Shelf	The water area of the cell that lies within the shelf zone (0–200 m depth), based on min/max elevation and proportion in depth zone	*km*^2^	2019		CSV	AquaMaps HCAF V7^112^	
Slope	The water area of the cell that lies within the slope zone (>200–4000 m depth), based on min/max elevation and proportion in depth zone.	*km*^2^	2019		CSV	AquaMaps HCAF V7^112^	
Abyssal	The water area of the cell that lies within the abyssal zone (>4000 m depth), based on min/max elevation and proportion in depth zone.	*km*^2^	2019		CSV	AquaMaps HCAF V7^112^	
Tidal Range	Extent of tides in scaled discrete classes	m	2019		CSV	AquaMaps HCAF V7^112^	LOICZ Database^121^
Coral	Proportion of whole (even non-water) cell covered by corals	%	2019		CSV	AquaMaps HCAF V7^112^	UNEP World Atlas of Coral Reefs^122^
Estuary	Area covered by estuaries in the cell	*km*^2^	2019		CSV	AquaMaps HCAF V7^112^	
Seamount	Number of known seamounts attributed to the cell	—	2019		CSV	AquaMaps HCAF V7^112^	
PWater	Proportion of water in each cell	%	2019		CSV	AquaMaps HCAF V7^112^	
Cell Area	The total area inside the cell in square kilometers, based on WGS84 and Miller cylindrical projection	*km*^2^	2019		CSV	AquaMaps HCAF V7^112^	
Sediment Thickness	Sediment thickness map obtained by combining high-resolution oceanic and tectonic maps with manually digitalised information	km	1997		NetCDF	Coro & Trumpy (2020)^118^	Laske (1997)^123^
Earth heat flow	Global Heat Flow: heat flow distribution map that represents the underground thermal state mainly affected by deep geological processes (i.e. radioactive decay of elements, tectonic setting, conduction etc.)	mW *m*^2^	2013		NetCDF	Coro & Trumpy (2020)^118^	Davies (2013)^124^
Distance from Earth Convergent Lines	Earth’s crust plates with subduction activity (convergent lines)	decimal degrees	2019		NetCDF	Coro & Trumpy (2020)^118^	United States Geological Survey data^125–127^
Distance from Earth Diffuse Lines	Earth’s crust plates with same relative motion (diffuse lines)	decimal degrees	2019		NetCDF	Coro & Trumpy (2020)^118^	United States Geological Survey data^125–127^
Distance from Earth Ridge Lines	Earth’s crust plates with ridges formation (ridge lines)	decimal degrees	2019		NetCDF	Coro & Trumpy (2020)^118^	United States Geological Survey data^125–127^
Distance from Earth Transform Lines	Earth’s crust plates with mutual sliding in opposite direction (transform lines)	decimal degrees	2019		NetCDF	Coro & Trumpy (2020)^118^	United States Geological Survey data^125–127^
Earthquake Density	Average earthquake density	number per cell	1900–2008		NetCDF	Coro & Trumpy (2020)^118^	Centennial Earthquake Catalog data^128,129^
Earthquake Depths	Average earthquake depths	km	1900–2008		NetCDF	Coro & Trumpy (2020)^118^	Centennial Earthquake Catalog data^128,129^
Earthquake Magnitudes	Average earthquake magnitudes	Ms	1900–2008		NetCDF	Coro & Trumpy (2020)^118^	Centennial Earthquake Catalog data^128,129^
Groundwater Resources	Groundwater and recharge map that represents large sedimentary basins suited for groundwater exploitation	mW *year*^−1^	2011		NetCDF	Coro & Trumpy (2020)^118^	World-wide Hydrogeological Mapping and Assessment Programme^130^

**Table 4:** Data of world population and marine-region parameters at 0.5° resolution at the global scale available in our repository, with indication of the related primary and secondary sources.

World population and marine-region parameters - 0.5° resolution global-scale data							
Parameter name	Description	Unit of measurement	Years or temporal aggregation	Climatic forecast scenarios	Original File Format	Primary source	Secondary sources
World population	World population density	persons per *km*^2^	2017		ESRI-GRID (ASC)	Coro (2020)^115^	Center for International Earth Science Information Network (gpwv4)^131^
EEZ	Exclusive Economic Zone (EEZ) in which the cell falls	—	2019		CSV	AquaMaps HCAF V7^112^	Marine Regions^39^
LME	Large Marine Ecosystem (LME) in which the cell falls	—	2019		CSV	AquaMaps HCAF V7^112^	Marine Regions^39^
MEOW	Marine Eco-regions of the World (MEOW) in which the cell falls	—	2019		CSV	AquaMaps HCAF V7^112^	Marine Regions^39^
MPA	Proportion of cell falling in a marine protected area	0–1 fraction	2019		CSV	AquaMaps HCAF V7^112^	

The earliest year involved in our collection is 1950. Forecasts are available for 2050 and 2100 under the Representative Concentration Pathway^25^ (RCP) scenarios 2.6 (63 data), 4.5 (162 data), and 8.5 (162 data), and the A2 Special Report on Emissions Scenarios^26^ (SRES) defined by the Intergovernmental Panel on Climate Change (IPCC) (180 data). These scenarios represent future greenhouse gas emission conditions and future societal development hypotheses. Temporal aggregation is annual for 196 data and monthly for 369 data (from January 2017 to March or May 2021, depending on the parameter). With a temporal coverage of ~10 years, between 1950 and 2100, our data are unsuited for running long-term continuous time series analyses. However, they are suited for creating long-term snapshots of ecological, environmental, and ecosystem models. Moreover, they allow for continuous time series analyses between 2016 and 2020 yearly (over 5 years, as demonstrated in this paper) and between 2017 and 2021 monthly (over 53 months), which are suited for finding evidence of inter-annual and inter-month variations and climate change-related variations^14,27^.

We checked the data against their primary sources for consistency. Moreover, we used a subset of annual data between 2016 and 2020, specifically created for this publication, to conduct a spatiotemporal analysis. This analysis confirmed similarities and discrepancies between the focus regions highlighted by independent studies (as indicated in the section “Technical Validation”), along with the parameters primarily responsible for the similarities.

## Methods

This section explains all workflow steps depicted in Fig. 1.

### Data

As the first workflow step, we collected data from the primary sources listed in Tables 1–4, which included:

1. Historical annual environmental data used by the AquaMaps ecological niche models and additional information attached to the AquaMaps authority files,
2. Re-processed or novel data attached to Italian National Research Council publications on marine science, Earth science, and epidemics,
3. Annual and monthly environmental data for the AquaMaps environmental parameters produced by the Quantitative Aquatics (Q-quatics) non-governmental organisation.

The data specifically produced for the present publication are the sea parameters reported in Table 1 with an asterisk. The re-distribution of the data was compliant with the primary and secondary source policies for the type of data re-processing we undertook. All data were globally distributed geospatial rasters; some were defined on marine areas only as that was appropriate for the ecological models of GES and EcoScope the datasets were used for. The data were defined on squared areas, with sides equal to the spatial resolution. Overall, the parameters involved were:

1. Sea-bottom and sea-surface dissolved oxygen, salinity, and temperature
2. Sea net primary production
3. Sea ice concentration
4. Average, minimum, maximum sea depth
5. Average, minimum, maximum elevation
6. Distance of a square marine area from land and its fraction covered by water
7. The characterization of each data cell in terms of which Large Marine Ecosystem (LME), Exclusive Economic Zone (EEZ), Marine Ecoregions of the World (MEOW), and Major Ocean Basins they belong to, and whether or not it sits in a Marine Protected Area (MPA)
8. Number of islands
9. Water area that lies within the shelf, slope, and abyssal zones
10. Tidal range extension
11. Coral density
12. Estuary and seamount presence
13. Carbon dioxide flux at soil surface
14. Air surface temperature
15. Precipitation
16. Difference between air surface temperature and sea surface temperature
17. World population density
18. Sediment thickness
19. Atmospheric concentration of methane and nitrous oxide
20. Earth heat flow
21. Distance from crust plates
22. Earthquake density, depth, magnitude
23. Groundwater resources

After the data collection phase, we harmonised all global-scale data from their primary sources’ geodetic systems to the same global-scale grid and projection, i.e., the WGS 84-EPSG:4326 geodetic system with equirectangular projection. We set two square grids for the data, at 0.5° and 0.1° depending on the original resolutions. The original files had heterogeneous formats, from raw text (CSV, XYZ) to more structured formats (NetCDF, ESRI-GRID). All files were first aligned to the same grid and checked for inconsistency and offset by comparing each grid point with the expected original data value. Eventually, they were converted to the ESRI-GRID ASCII format (ASC)^28^. ESRI-GRID is a standard format approved by the Open Geospatial Consortium (OGC), a worldwide community that assesses standards and protocols to improve access to geospatial data. This format allows for inspecting the data with text processing software as well as visualising them with commonly used Geographic Information System (GIS) software (e.g., QGIS^29^, and ArcGIS^30^). The format is also the most frequently accepted by ecological niche modelling and ecosystem modelling software (e.g, MaxEnt^31^ and Ecopath with Ecosim^22,32–34^) and most programming languages have libraries for parsing it^35,36^. In our harmonisation and standardisation workflow, one ASC file corresponds to one parameter in a specific year (or month) and location. This correspondence makes the files easily convertible into other formats (e.g., in NetCDF format through the GDAL software^37^). Overall, the ESRI-GRID ASCII format was optimal for our collection’s scope of supporting ecological and ecosystem models and climatic analyses.

All data were also cut out on 8 European marine areas of particular economic or ecosystem importance, identified by the EcoScope European Project community of practice. These areas (hereafter named *focus regions*) were:

1. The global-scale
2. The Adriatic Sea
3. The Aegean Sea
4. The Baltic Sea
5. The Bay of Biscay
6. The Black Sea
7. The Levantine Sea
8. The North Sea
9. The Western Mediterranean Sea

The areas were geographically identified according to the corresponding marine eco-region (Adriatic, Aegean, Baltic, Levantine, the North Sea) or International Hydrographic Organization region (Bay of Biscay, the Black Sea, Western Mediterranean Sea)^38,39^.

The temporal coverage of our data collection is of ~10 years within the period 1950–2100. Forecasts for 2050 and 2100 are available under different greenhouse gas emission scenarios, i.e., RCP 2.6 (low emission), 4.5 (medium emission), and 8.5 (high emission), although the RCP 2.6 scenario was not available for 2050. Moreover, some forecasts for the IPCC SRES A2 scenario (which hypothesises a future of independent, self-reliant nations with constantly increasing population and regionally diversified economic development, slow technological change, and worldwide use of nuclear energy) were also available and included in the collection.

Data harmonisation for text files was conducted through a dedicated Java process^40^ that managed the different formats, aligned the data to a resolution-specific grid, and finally produced one ASC file. As for primary sources with NetCDF and ESRI-GRID formats, we performed manual checking, alignment, and band extraction through QGIS. Conversion to ASC format was done through GDAL. No-data locations were all assigned a default −9999 value, specified in the ASC file header through the NODATA attribute, which makes it automatically interpreted and used by GIS software for consumption and visualisation. Data with non-homogeneous resolution over longitude and latitude were homogenised through nearest-neighbour and bilinear interpolation separately, via QGIS. Earthquake and high-resolution temperature and precipitation data were left to their original aggregated temporal range to represent an aggregated reference of a recent past.

### Newly produced data and time series analysis

We conducted a spatiotemporal analysis on the focus regions to find evidence of similarities between parameter trends over the years. Then we checked for agreement with outputs of other studies as a further data validation. We focussed this analysis on a data collection subset containing newly produced data at 0.1° resolution, annually aggregated from 2016 to 2020 (Table 1). We selected these data because they were not previously explicitly validated in other publications, and were thus differentiated from the other data whose content was instead validated in other publications^12,16,17,41–44^.

**Fig. 2:**
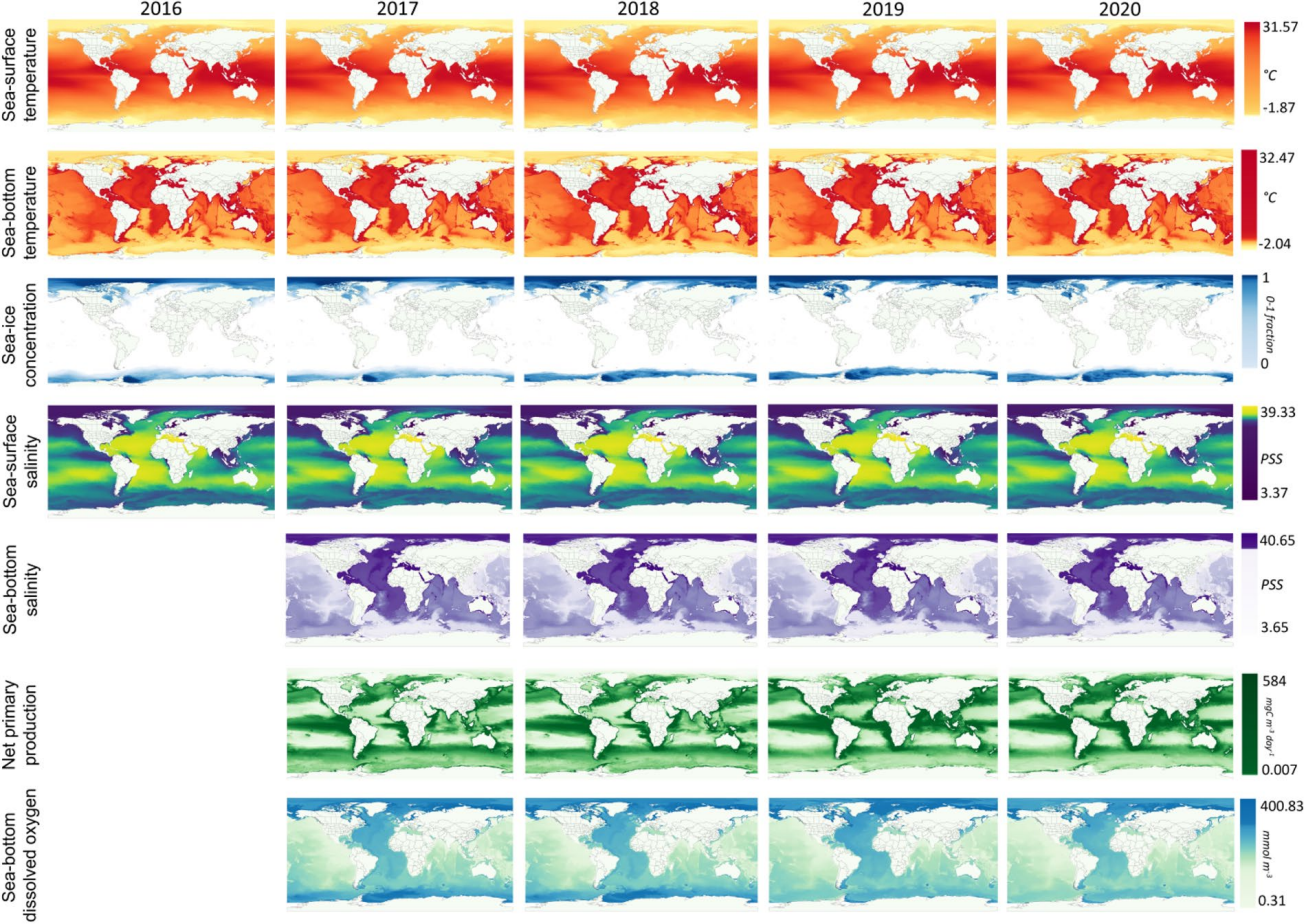
Comparison between the distributions of the environmental parameters used for time series and habitat analyses. The displayed maps have a global-scale 0.1° resolution.

The selected data were the following (Fig. 2):

1. Sea-surface temperature
2. Sea-bottom temperature
3. Sea-ice concentration
4. Sea-surface salinity
5. Sea-bottom salinity
6. Sea net primary production
7. Sea-bottom dissolved oxygen

These parameters are generally used by the AquaMaps ecological niche models^41^ that assume they include sufficient information to assess global species presence^16^. It is important to note that 2016 data were not available for sea-bottom salinity, sea net primary production, and sea-bottom dissolved oxygen.

We used ocean products from the Copernicus Marine Service^45^ to produce the new data for the seven environmental parameters above. NetCDF data for mean monthly sea surface and bottom temperature, sea surface and sea bottom salinity, and sea ice concentration were re-processed based on the Global Ocean 1/12° Physics Analysis and Forecast 001–024 monthly dataset^46^, that natively used the WGS 84-EPSG:4326 geodetic system and equirectangular projection and had 0.083° spatial resolution. Mean monthly data for net primary production and dissolved oxygen were obtained from two temporally complementary datasets: the Global Ocean Biogeochemistry Analysis and Forecast 001–028 monthly dataset^47^ (in WGS 84-EPSG:4326 geodetic system and projection) and the Global Ocean Biogeochemistry Hindcast 001–029 monthly dataset^47^ (in ETRS 89-EPSG:4258 geodetic system and projection), both having a spatial resolution of 0.25°. Global monthly data for sea surface and bottom temperature, sea surface and bottom salinity, sea ice concentration were rasterized and resampled using the R-Terra package^48^, upscaling to 0.1° spatial resolution using bilinear interpolation. Net primary production and dissolved oxygen data were all reprojected to WGS 84-EPSG:4326 prior to rasterization and resampled by downscaling to 0.1° spatial resolution using bilinear interpolation. We carried out this process only on one depth layer (either surface or bottom) for all parameters, except for sea bottom salinity and bottom dissolved oxygen. These two parameters required the resampling of up to 72 depth levels (0.5 m to 5902 m) per month, then extracting data at the maximum depth layer per 0.1° grid cell before compiling them into corresponding monthly sea bottom salinity and sea bottom dissolved oxygen raster layers. We then used the resampled monthly rasters to compute the annual means for each of the seven parameters. The annual mean data were saved as GeoTIFF and CSV formats and were manually inspected for exact correspondence through coordinate mapping in ArcGIS^30^. Cases where precedent resampling to 0.1° spatial resolution had yielded marginal rows (along 89.95°N or 76.95°S) or a marginal column (along 179.95°E) with missing data were resolved by copying parameter values directly from the neighboring row or column. This approach was considered reasonable in view of the spatial resolution of the data. The final outputs were exported as CSV files and underwent the data harmonisation and standardisation process depicted in Fig. 1.

To validate the data, annual average values per region were first extracted and visualised for each parameter to compare trends across all regions (section “Technical Validation”). Moreover, each region was characterised through its associated parameter time series. Average time series 0-lag cross-correlation was used for numerical comparison. Specifically, it was calculated per parameter across all focus regions, and per region across all parameters. These analyses highlighted general and regional parameter time series similarities. Confirmation of these similarities with that seen in other scientific studies was used to assess the reliability of the data in representing valid ecological macro-patterns.

### Habitat representativeness score

Parameter time series cross-correlation might indicate that two regions were subject to similar average parameter variations. This condition might correspond to similar habitats over time in geographically connected regions if the parameters have similar ranges and distributions. A species’ ecological niche is, mathematically, the space within a hyper-volume in a vector space of environmental parameters associated with the species’ proliferation. Understanding general habitat similarity between two regions is equivalent to assessing the similarity between the parameter hyper-volumes over the two regions, independently of the species. This assumption is reasonable if the involved parameter set is complete enough for ecological niche modelling. Correlated region-specific parameter time series do not necessarily indicate similar habitats, because parameter distributions’ similarity and geographic reachability are also required. Habitat similarity, which depends on annual parameters’ distributions, can also change over the years and is thus complementary information with respect to time series correlations. Habitat dissimilarity after a specific year, unnecessarily corresponding to lower time series cross-correlation, likely indicates that an abrupt event made two regions different.

We conducted habitat similarity analysis over the years between our focus regions to study these variations and search for confirmations in other studies. Specifically, we used the Habitat Representativeness Score (HRS)^49^ to measure habitat similarity. HRS is an algorithm based on Principal Component Analysis (PCA)^50^ that measures the overall difference of the data distributions between two regions, across the largest data variance directions (principal components). HRS has been used to understand the principal environmental drivers of species presence in distant regions^51^ and to assess ecological survey completeness^49^. The algorithm works with two inputs: a reference region *A* and a test region *B*. As the output, it calculates a score interpretable as the representativeness of habitat *B* by habitat *A* (*HRS*(*A, B*)). Each region is characterised through vectors of environmental parameters. PCA is conducted on the reference region (*A*) vectors to extract major data variance axes (i.e., the principal components). An optional threshold, set on the principal components’ eigenvalues, can restrict the comparison to the largest variance axes. In our validation experiment, we selected components covering up to 95% of the total data variance. Then, the normalised data frequency distribution of the vectors on each axis is calculated and subdivided into equal-frequency bins. The region *B* vectors are then projected onto the principal components of region *A*. The *B* parameter frequencies over the *A* principal components are calculated across the same bins estimated for *A*. Finally, the pairwise differences between the bin frequencies are calculated for all principal components. The HRS is the sum of these pairwise differences. Since bin frequencies sum to 1 on each axis, the HRS ranges from 0 to the number of principal components (*N*), with *N* representing completely different habitats and 0 perfect habitat similarity.

We calculated a pairwise HRS matrix to discover significant habitat similarities between the focus regions. However, HRS is an asymmetric function by construction because PCA conducted on region *A* and projected on *B* likely gives different results than PCA conducted on region *B* and projected on *A*. One possible estimation of the overall HRS between *A* and *B* is the mean between *HRS*(*A,B*) and *HRS*(*B,A*)^52^. This choice also makes the HRS matrix symmetric and facilitates the similarity analysis. Therefore, we used average HRS as the region-pair score. For each region, we standardised the scores by dividing the value by the total HRS range. We finally assessed as “similar” those region pairs emerging from the standardisation by more than 10%. We repeated this analysis for all annual data between 2016 and 2020 to study habitat similarity stability over the years between the focus regions. Finally, we verified evidence of the detected similarity stability and instability in other studies.

### Detecting major habitat similarity drivers

Using PCA over the focus regions’ parameters allowed for consideration of the variables that primarily contributed to the largest principal components and thus to the HRS^50,53^. In particular, the PCA principal axes (eigenvectors) and their eigenvalues allow for defining *loading* vectors as $loading=eigenvector\cdot \sqrt{eigenvalue}$. Each *loading* is a vector containing as many elements as the number of original environmental parameters, and there is one loading for every PCA axis. The loading vector elements represent the original environmental parameters’ contributions to the corresponding PCA axis (*weights*). Keeping only the major PCA axes (i.e., those with the largest eigenvectors) allows for the analysis to be focussed on the largest data variance and exclude noise. Average parameter weight across the loadings measures the average contribution to the principal components by each parameter and thus the contribution to the HRS. Therefore, the parameters with the largest average weights are the major drivers of the estimated HRSs and thus of the detected similarities. For the present loadings analysis, we selected the principal components covering up to 95% of the total data variance and the environmental parameters with a non-zero positive average weight.

## Data Records

**Fig. 3:**
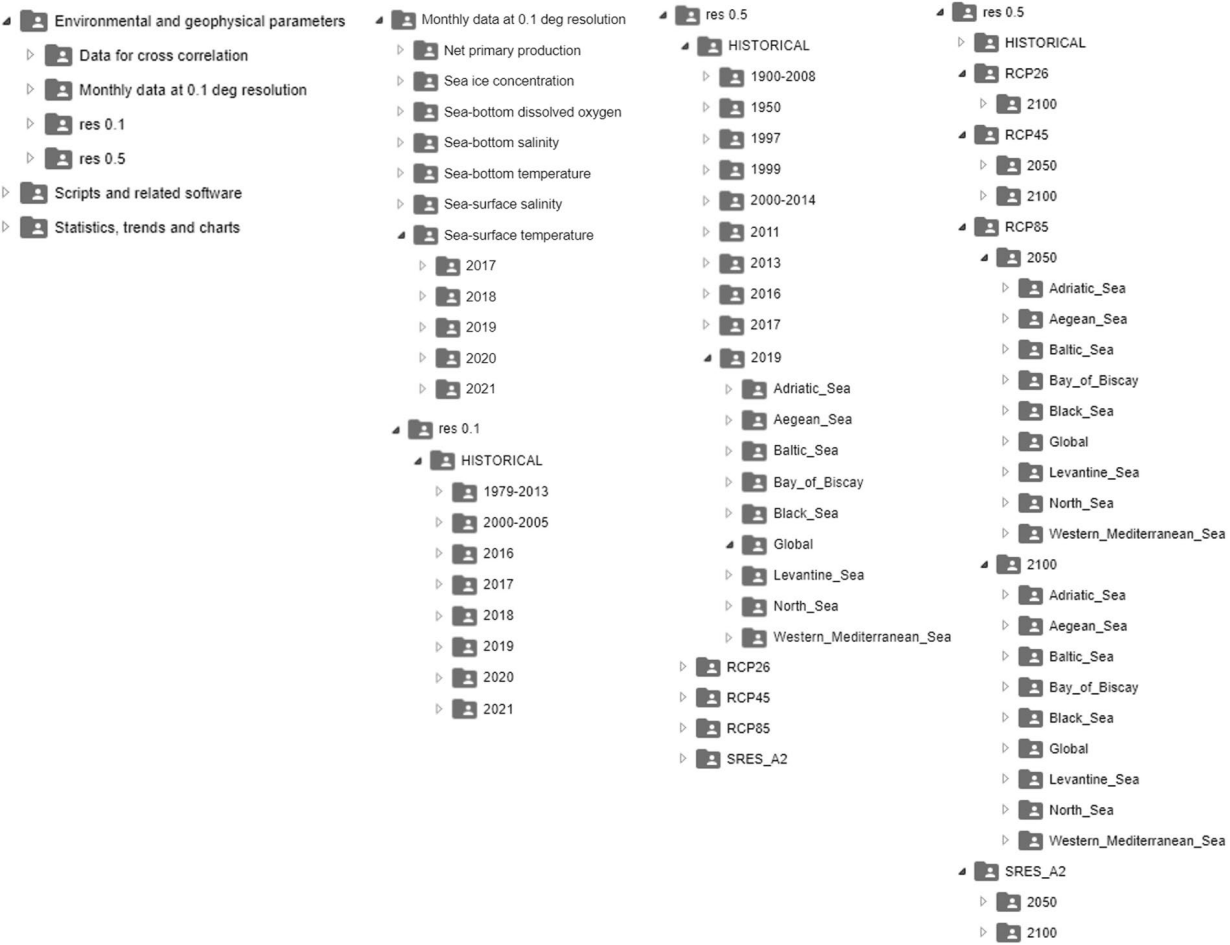
Folder structure of our data repository.

We made the data available on a public-access Figshare repository^54^. The collection is composed of 6 datasets. The principal datasets are *“Environmental Geophysical Marine Socioeconomic parameters at 0.1° and 0.5° resolutions”* and *“Monthly data at 0.1° resolution”*. Internally, they are structured with a folder hierarchy that optimises search time for an ecological niche modelling expert (Fig. 3). The first dataset separates 0.5° and 0.1° spatial resolution files in two main folders. The 0.5° resolution folder contains one sub-folder each for RCP 2.6, 4.5, and 8.5, the IPCC SRES A2 forecast scenario, and historical data (named HISTORICAL). Each sub-folder is organised by year. For example, the RCP 4.5, RCP 8.5, and IPCC SRES A2 folders contain the 2050 and 2100 sub-folders. The RCP 2.6 folder contains only the 2100 folder. The HISTORICAL data folder contains year-specific sub-folders from 1950 to 2019 and two additional folders for the 1900–2008 and 2000–2014 temporal aggregations. Each annual sub-folder contains one sub-folder for each focus region (9 total), which in turn contains the ESRI-GRID parameter files with the specific resolution, scenario, time reference, and region corresponding to the file path and the metadata. Each file name contains information to reconstruct the path. For instance, *Sea-surface_temperature_res_05_annual_years_*2019*_Clim_scen_historical_regional_Adriatic_Sea.asc* indicates a file containing annual-aggregated sea-surface temperature data, at 0.5° resolution, in 2019, within the HISTORICAL data sub-set, and cut out on the Adriatic Sea. The 0.1° annual data root folder has the same structure as the 0.5° root folder but contains only historical data. The years involved in our data collection are: 1950, 1997, 1999, 2011, 2013, 2016, 2017, 2018, 2019, 2050, and 2100. Some files, e.g., those of gas concentration of methane and nitrous oxide, have a variant file with the “bilinear” attribute in the file name to indicate that bilinear interpolation was used instead of nearest neighbour to homogenise coordinate resolutions.

The adopted folder structure allows an ecological niche modelling expert to find aligned files in one folder and directly use them in modelling software like MaxEnt^31^, e.g., to quickly model a species’ distribution at a 0.5° spatial resolution in 2019 in the Adriatic.

The monthly dataset has a folder structure organised by parameter name. Each parameter folder contains one sub-folder for each year, which in turn contains monthly ESRI-GRID files. This structure is conceived to facilitate monthly parameter analyses.

The complete file collection contains 2132 files. Global-scale data are 565; 154 have 0.5°resolution and 411 have 0.1° resolution. Among the 0.1° resolution data, 369 have a monthly aggregation and 196 an annual aggregation. Forecasts are available for 2050 and 2100, and overall include 63 files for RCP 2.6 (only in 2100), 162 for RCP 4.5, 162 for RCP 8.5, and 180 for IPCC SRES A2.

Additional datasets in the collection (in the “Statistics, trends, HRS, PCA-loadings, and charts” and “File list and statistical properties” datasets) contain summary tables and charts with standard statistics (mean, standard deviation, geometric mean, log-normal standard deviation), cross-correlations, HRS estimates, and PCA loadings that we used for the technical validation. The Figshare repository also contains all R scripts, Java software links, and references to the programs used to conduct the technical validation (in the “Scripts and related software” dataset).

## Technical Validation

### Consistency with respect to the original data

Each produced ESRI-GRID file was defined on a regular spatial grid. Therefore, as a first consistency check, we exhaustively verified that all grid data corresponded to the expected original data. In particular, we systematically sampled from each ESRI-GRID file and pairwise checked if the samples corresponded, through coordinate mapping, to the expected values in the original dataset. As for interpolated coordinates, the nearest neighbour value in the original file was taken as the validation reference. This operation allowed us to detect conversion and misalignment errors, which we later adjusted for exact correspondence with the original files. We conducted this operation with a specific Java-based program for text files^40^, and with QGIS and GDAL for NetCDF and ASC files. General content validation was also conducted by manually checking if the means, standard deviations, geometric means, and log-normal standard deviations (for positive-defined variables) of all files fell in the expected ranges. A summary table of statistics for all files is available in our repository^55^. The script for calculating this table is available in the “Scripts and related software” dataset^54^.

The quality of the data from our previous studies was already verified in the original publications (referred in Tables 1–4), and in other additional publications^12,16,17,41–44^. Therefore, we technically validated these files by checking their ESRI-GRID version consistency with the original files.

As for the newly generated data, we assessed their quality by searching for evidence of the inferred trends and similarities in other studies (explained in the following sections).

### Time series cross-correlation analysis

**Fig. 4:**
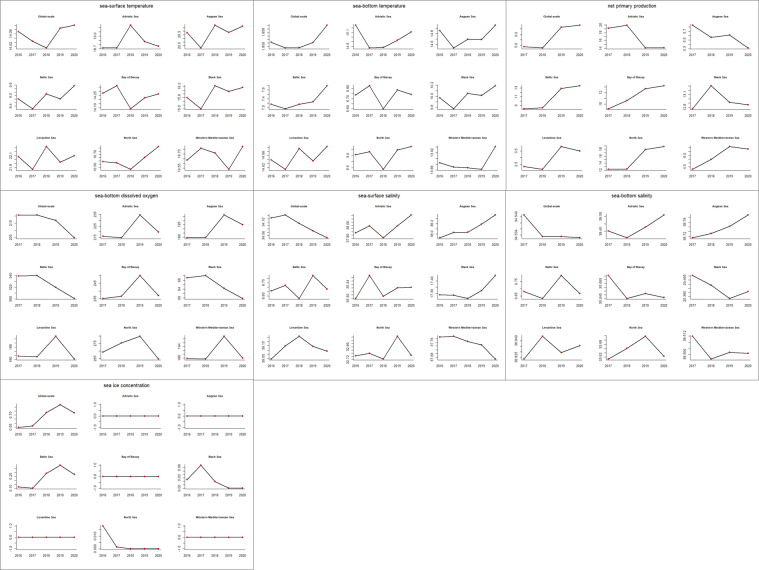
Time series of average environmental parameter values per focus region. The reported parameters are those used for cross-correlation and habitat analyses and have 0.1° spatial resolution.

**Fig. 5:**
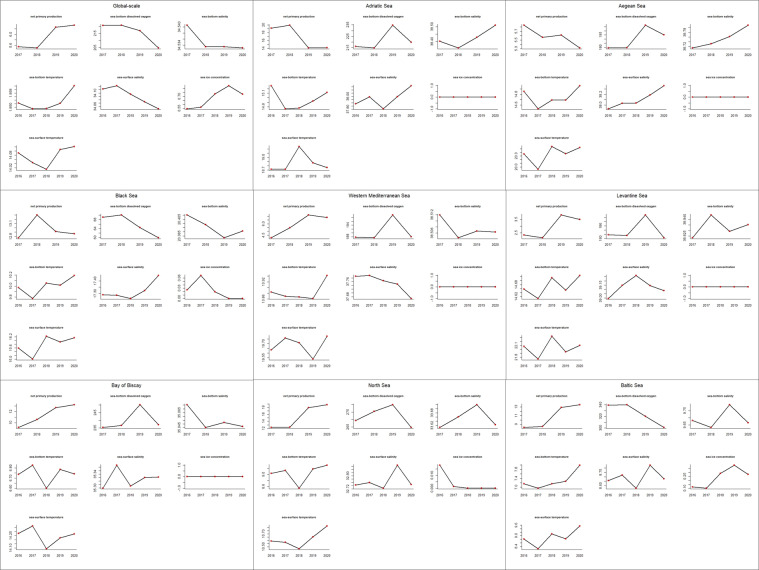
Characterisation of our focus regions through environmental parameters’ time series. The reported parameters are those used for cross-correlation and habitat analyses and have 0.1° spatial resolution.

We produced two charts to visually summarise (i) the parameter time series over the focus regions and (ii) the focus regions’ characterisation in terms of parameter variations (Figs. 4, 5). Moreover, in Table 5, we summarised the parameter trends confirmed by other studies. For each parameter, we also reported the percentage of regions (over the total nine regions) for which we found studies confirming or explaining the trends. The parameter charts highlight an inconstant trend of sea-surface and -bottom temperature across the regions. Moreover, sea temperature had a general increasing trend at the global scale (more than linearly for sea-bottom temperature), which several other studies have confirmed in the last decades^56,57^. Net primary production presented a globally increasing trend, in agreement with other studies^58,59^, and an overall decreasing trend in the Adriatic, Aegean, and the Black Sea also highlighted by other studies^14,60,61^. Sea-bottom dissolved oxygen presented a non-linear global-scale decrease in 2020 in all regions, also confirmed by other studies^62–64^. Sea-surface and -bottom salinity had a globally decreasing trend in several regions, probably because of ice melting and climate change-related freshwater fluxes^65–67^. A salinity increasing trend occurred for the Black Sea, also observed by another study^68^. A significant sea ice concentration variation occurred in the Baltic Sea, with an increasing trend up to 2019 followed by a lower value in 2020, reflecting the global trend. A decrease occurred in the Black Sea and the North Sea. These observations agree with other studies^69–72^.

**Table 5:** Summary table of the trends observed in our data that agreed with other studies, and the percentage of regions (over the total 9 regions) for which we found trend confirmation in other studies.

Parameter name	Confirmed trends from other studies	Confirmation percentage across the regions
Sea-surface temperature	Averagely increasing trend	56%
Sea-bottom temperature	Averagely increasing trend	89%
Net primary production	Averagely increasing trend, with local decrease in the Adriatic, Aegean, and the Black Sea	100%
Sea-bottom dissolved oxygen	Global decreasing trend, with decrease in 2020 in all regions	100%
Sea-surface salinity	Averagely decreasing trend, with increase in the Black Sea regions	67%
Sea-bottom salinity	Averagely decreasing trend	56%
Sea ice concentration	Averagely increasing trend up to 2019 and then decreasing; overall decrease in the North Sea and Black Sea regions	100%

Averaging time series cross-correlations across the areas revealed overall similarities between the parameter trends. We highlighted direct and inverse correlations in the comparison matrix to study the significant similarities. Specifically, we studied the direct and inverse correlations being at least moderate^73^, i.e., higher than 30% or lower than −30% (Table 6).

**Table 6:** Pairwise cross-correlations between parameter time series (upper table) with the indication of the focus regions where the cross-correlations were significantly direct (+) or inverse (−) (lower table).

	net primary production	sea-bottom dissolved oxygen	sea-bottom salinity	sea-bottom temperature	sea-surface salinity	sea ice concentration	sea-surface temperature
**net primary production**		0.5%	**−32%**	5%	**−36%**	−3%	11%
**sea-bottom dissolved oxygen**	0.5%		19%	−22%	0.3%	−1%	**−37%**
**sea-bottom salinity**	**−32%**	19%		*30%*	*65%*	11%	11%
**sea-bottom temperature**	5%	−22%	*30%*		24%	−6%	*64%*
**sea-surface salinity**	**−36%**	0.3%	*65%*	24%		−6%	9%
**sea ice concentration**	−3%	−1%	11%	−6%	−6%		−6%
**sea-surface temperature**	11%	**−37%**	11%	*64%*	9%	−6%	
	**net primary production**	**sea-bottom dissolved oxygen**	**sea-bottom salinity**	**sea-bottom temperature**	**sea-surface salinity**	**sea ice concentration**	**sea-surface temperature**
**net primary production**			**(−)Global-scale, (−)Adriatic Sea, (−)Aegean Sea, (+)Baltic Sea, (−)Bay of Biscay, (+)North Sea, (−)Western Mediterranean Sea**		**(−)Global-scale, (−)Adriatic Sea, (−)Aegean Sea, (+)Baltic Sea, (−)Bay of Biscay, (−)Black Sea, (−)Levantine Sea, (+)North Sea, (−)Western Mediterranean Sea**		
**sea-bottom dissolved oxygen**							**(−)Global-scale, (−)Baltic Sea, (−)Black Sea, (−)North Sea, (−)Western Mediterranean Sea**
**sea-bottom salinity**	**(−)Global-scale, (−)Adriatic Sea, (−)Aegean Sea, (+)Baltic Sea, (−)Bay of Biscay, (+)North Sea, (−)Western Mediterranean Sea**			*(−)Global-scale, (+)Adriatic Sea, (+)Aegean Sea, (+)Bay of Biscay, (−)Black Sea, (+)Levantine Sea*	*(+)Global-scale, (+)Adriatic Sea, (+)Aegean Sea, (+)Baltic Sea, (+)Bay of Biscay, (−)Black Sea, (+)Levantine Sea, (+)North Sea, (+)Western Mediterranean Sea*		
**sea-bottom temperature**			*(−)Global-scale, (+)Adriatic Sea, (+)Aegean Sea, (+)Bay of Biscay, (−)Black Sea, (+)Levantine Sea*				*(+)Global-scale, (−)Adriatic Sea, (+)Aegean Sea, (+)Baltic Sea, (+)Bay of Biscay, (+)Black Sea, (+)Levantine Sea, (+)North Sea, (+)Western Mediterranean Sea*
**sea-surface salinity**	**(−)Global-scale, (−)Adriatic Sea, (−)Aegean Sea, (+)Baltic Sea, (−)Bay of Biscay, (−)Black Sea, (−)Levantine Sea, (+)North Sea, (−)Western Mediterranean Sea**		*(+)Global-scale, (+)Adriatic Sea, (+)Aegean Sea, (+)Baltic Sea, (+)Bay of Biscay, (−)Black Sea, (+)Levantine Sea, (+)North Sea, (+)Western Mediterranean Sea*				
**sea ice concentration**							
**sea-surface temperature**		**(−)Global-scale, (−)Baltic Sea, (−)Black Sea, (−)North Sea, (−)Western Mediterranean Sea**		*(+)Global-scale, (−)Adriatic Sea, (+)Aegean Sea, (+)Baltic Sea, (+)Bay of Biscay, (+)Black Sea, (+)Levantine Sea, (+)North Sea, (+)Western Mediterranean Sea*			

Text style indicates overall significant direct (italics) or inverse (bold) correlations.

This analysis revealed the following time series similarities in the analysed time frame:

- Net primary production, on average, was inversely correlated with sea-bottom and -surface salinity, especially at the global scale and in the Adriatic, Aegean, Bay of Biscay, Black Sea, Levantine, and Western Mediterranean. These observations agree with those of other studies;^14,74–77^
- Sea-bottom salinity was generally positively correlated with sea-surface salinity in most seas except for the Black Sea, due to peculiar deep and shallow thermohaline dynamics^78^. It was also positively correlated with sea-bottom temperature in the Adriatic, Aegean, Bay of Biscay, and Levantine, as can also be inferred by other studies^79–82^;
- Sea-bottom dissolved oxygen was inverse-correlated with sea-surface temperature at the global scale, and in the Baltic, Black Sea, North Sea, and Western Mediterranean, as inferable also by other studies;^63,64,78,83–85^
- Sea-ice concentration had no significant correlation with the other parameters.

Repeating the same analysis by focus region highlighted the following moderate^73^ correlations (Table 7):

- The global scale had similar trends to those of the Baltic Sea (because of a similar ice concentration trend), Bay of Biscay, and Western Mediterranean because they shared averagely increasing net primary production and temperature trends^56–59^. Conversely, the global scale had different trends with respect to those of the Adriatic due to different parameter signal-phases;^14,86^
- The Adriatic Sea time series were correlated with those of the Aegean Sea through similar trends of all parameters^87^;
- The Aegean Sea time series were correlated with those of the Levantine Sea through similar sea-bottom dissolved oxygen and sea-bottom and -surface temperature trends^87^;
- The Baltic time series were correlated with those of the North Sea through all parameters except sea-ice concentration^88,89^;
- The Bay of Biscay had similar trends to those of the North Sea and the Western Mediterranean through net primary production, sea-bottom dissolved oxygen, temperature (only for the North Sea), and sea-bottom salinity (only for the Western Mediterranean) because of constant inter-connected water mass flow exchange^90,91^;
- The Black Sea had a standalone characterisation and non-significant cross-correlation with the other regions^78^;

**Table 7:** Average time series cross-correlations between focus regions (upper sub-table) with the indication of the environmental parameters on which the cross-correlations were significantly direct (+) or inverse (−) (lower table). Text style indicates overall significant direct (italics) or inverse (bold) correlations.

	Global-scale	Adriatic Sea	Aegean Sea	Baltic Sea	Bay of Biscay	Black Sea	Levantine Sea	North Sea	Western Mediterranean Sea
**Global-scale**		−30%	−24%	*57%*	*36%*	10%	11%	23%	*50%*
**Adriatic Sea**	**−30%**		*66%*	0%	−6%	22%	5%	−7%	−2%
**Aegean Sea**	−24%	*66%*		11%	−19%	16%	*30%*	7%	−8%
**Baltic Sea**	*57%*	0%	11%		10%	17%	22%	*50%*	26%
**Bay of Biscay**	*36%*	−6%	−19%	10%		−11%	−6%	*39%*	*45%*
**Black Sea**	10%	22%	16%	17%	−11%		9%	−4%	0%
**Levantine Sea**	11%	5%	*30%*	22%	−6%	9%		20%	24%
**North Sea**	23%	−7%	7%	*50%*	*39%*	−4%	20%		25%
**Western Mediterranean Sea**	*50%*	−2%	−8%	26%	*45%*	0%	24%	25%	
	**Global-scale**	**Adriatic Sea**	**Aegean Sea**	**Baltic Sea**	**Bay of Biscay**	**Black Sea**	**Levantine Sea**	**North Sea**	**Western Mediterranean Sea**
**Global-scale**		**(−)net primary production, (+)sea-bottom temperature, (−)sea-surface salinity, (−)sea-surface temperature**		*(+)net primary production, (+)sea-bottom dissolved oxygen, (+)sea-bottom temperature, (+)sea ice concentration, (+)sea-surface temperature*	*(+)net primary production, (+)sea-bottom salinity, (+)sea-surface temperature*				*(+)net primary production, (+)sea-bottom salinity, (+)sea-bottom temperature, (+)sea-surface salinity*
**Adriatic Sea**	**(−)net primary production, (+)sea-bottom temperature, (−)sea-surface salinity, (−)sea-surface temperature**		*(+)net primary production, (+)sea-bottom dissolved oxygen, (+)sea-bottom salinity, (+)sea-bottom temperature, (+)sea-surface salinity, (+)sea-surface temperature*						
**Aegean Sea**		*(+)net primary production, (+)sea-bottom dissolved oxygen, (+)sea-bottom salinity, (+)sea-bottom temperature, (+)sea-surface salinity, (+)sea-surface temperature*					*(−)net primary production, (+)sea-bottom dissolved oxygen, (+)sea-bottom temperature, (+)sea-surface temperature*		
**Baltic Sea**	*(+)net primary production, (+)sea-bottom dissolved oxygen, (+)sea-bottom temperature, (+)sea ice concentration, (+)sea-surface temperature*							*(+)net primary production, (+)sea-bottom dissolved oxygen, (+)sea-bottom salinity, (+)sea-bottom temperature, (+)sea-surface salinity, (−)sea ice concentration, (+)sea-surface temperature*	
**Bay of Biscay**								*(+)net primary production, (+)sea-bottom dissolved oxygen, (−)sea-bottom salinity, (+)sea-bottom temperature, (+)sea-surface temperature*	*(+)net primary production, (+)sea-bottom dissolved oxygen, (+)sea-bottom salinity*
**Black Sea**									
**Levantine Sea**			*(−)net primary production, (+)sea-bottom dissolved oxygen, (+)sea-bottom temperature, (+)sea-surface temperature*						
**North Sea**				*(+)net primary production, (+)sea-bottom dissolved oxygen, (+)sea-bottom salinity, (+)sea-bottom temperature, (+)sea-surface salinity, (−)sea ice concentration, (+)sea-surface temperature*	*(+)net primary production, (+)sea-bottom dissolved oxygen, (−)sea-bottom salinity, (+)sea-bottom temperature, (+)sea-surface temperature*				
**Western Mediterranean Sea**					*(+)net primary production, (+)sea-bottom dissolved oxygen, (+)sea-bottom salinity*				

The regions with higher cross-correlation were geographically connected regions that share sea-currents and partially overlap. Although the correlations generally do not correspond to habitat similarity, they might indicate similar area responses to inter-annual parameter variations and climate change^17^.

### Habitat similarities

To further explore if time-series cross-correlations were accompanied by habitat similarities, we calculated HRSs between the focus regions. We used a re-implementation of the HRS algorithm^92^, also available as a Web tool^93^ on the D4Science e-Infrastructure^94–98^. The HRSs were calculated on annual parameters from 2017 to 2020 (Table 8). The comparison was not reported for 2016 because HRS could not be calculated for all parameters. The global scale was excluded from the focus regions because calculating the HRS against much smaller areas would not have been meaningful due to the incommensurable data variabilities. HRSs were categorised as similar/dissimilar based on the threshold described in the Methods section. Numerical details are reported in the “Statistics, trends, HRS, PCA-loadings, and charts” dataset on our Fisgshare repository^54^.

**Table 8:** Habitat similarity highlights, based on the Habitat Representativeness Score algorithm, between the focus regions over the years. Checkmarks indicate significant habitat similarity; empty cells indicate non-significant similarity.

Highlight of pair habitat similarity over the years								
2020								
	Adriatic Sea	Aegean Sea	Baltic Sea	Bay of Biscay	Black Sea	Levantine Sea	North Sea	Western Mediterranean Sea
Adriatic Sea		✓						
Aegean Sea	✓							
Baltic Sea							✓	
Bay of Biscay								
Black Sea								
Levantine Sea								
North Sea			✓					
Western Mediterranean Sea								
**2019**								
	**Adriatic Sea**	**Aegean Sea**	**Baltic Sea**	**Bay of Biscay**	**Black Sea**	**Levantine Sea**	**North Sea**	**Western Mediterranean Sea**
Adriatic Sea		✓						
Aegean Sea	✓							
Baltic Sea							✓	
Bay of Biscay								
Black Sea								
Levantine Sea								
North Sea			✓					
Western Mediterranean Sea								
**2018**								
	**Adriatic Sea**	**Aegean Sea**	**Baltic Sea**	**Bay of Biscay**	**Black Sea**	**Levantine Sea**	**North Sea**	**Western Mediterranean Sea**
Adriatic Sea								
Aegean Sea								
Baltic Sea							✓	
Bay of Biscay								
Black Sea								
Levantine Sea								
North Sea			✓					
Western Mediterranean Sea								
**2017**								
	**Adriatic Sea**	**Aegean Sea**	**Baltic Sea**	**Bay of Biscay**	**Black Sea**	**Levantine Sea**	**North Sea**	**Western Mediterranean Sea**
Adriatic Sea								
Aegean Sea								
Baltic Sea							✓	
Bay of Biscay								
Black Sea								
Levantine Sea								
North Sea			✓					
Western Mediterranean Sea								

The HRS table indicates that habitat similarity occurred between the Aegean and Adriatic Seas only in 2019 and 2020 and between the North Sea and Baltic Sea from 2017 to 2020. These regions also have generally similar parameter trends and are geographically connected. Habitat dissimilarity between the Aegean and Adriatic seas in 2018 and 2017 corresponds to a known effect of an anticyclonic Bimodal Oscillating System regime that prevented eastern waters from entering the Adriatic in those years^86,99–101^. General habitat similarity between the North Sea and the Baltic Sea has also been highlighted by other studies, unless regime shifts occur^102–104^. This overall similarity is also demonstrated by the many fishery-targeted species living in both the areas, e.g., *Gadus morhua*, *Limanda limanda*, *Platichthys flesus*, *Pleuronectes platessa*, *Scophthalmus maximus*, *Scophthalmus rhombus*, and *Solea solea*.

As for the other focus regions, the similar time series trends in the previous section did not correspond to habitat similarity. Thus, these regions can present similar inter-annual parameter changes but dissimilar parameter distributions.

### Habitat similarity drivers

The extracted PCA loadings (Table 9) shed light on the parameters’ variability over the years and their contributions to HRSs. This analysis highlighted that the similarity between Adriatic and Aegean seas was mainly driven by the sea-bottom temperature distribution. In the Aegean Sea, this parameter had a higher weight in 2020 and 2019 than in 2018 and 2017, and its distribution resembled the one of the Adriatic Sea in 2020 and 2019. The parameter contribution rankings in 2018 and 2017 in the Aegean Sea changed with respect to 2020 and 2019, in correspondence of the anticyclonic Bimodal Oscillating System regime effect^86,99–101^.

**Table 9:** Contribution of the environmental parameters to the PCA loadings across the focus regions over the years.

Adriatic Sea			
2017	2018	*2019*	*2020*
Net Primary Production (77.1%)	Net Primary Production (81.6%)	Net Primary Production (74.3%)	Net Primary Production (77.7%)
**Sea-bottom temperature (22.9%)**	**Sea-bottom temperature (18.4%)**	**Sea-bottom temperature (25.7%)**	**Sea-bottom temperature (22.3%)**
Aegean Sea			
**2017**	**2018**	*2019*	*2020*
Sea-bottom dissolved oxygen (31.7%)	Sea-bottom dissolved oxygen (30.0%)	**Sea-bottom temperature (29.5%)**	**Sea-bottom temperature (26.4%)**
**Sea-bottom temperature (23.5%)**	**Sea-bottom temperature (22.8%)**	Sea-bottom dissolved oxygen (19.8%)	Sea-bottom dissolved oxygen (21.5%)
Sea-surface salinity (19.3%)	Sea-surface salinity (19.4%)	Sea-surface salinity (19.4%)	Sea-surface salinity (19.9%)
Sea-surface temperature (16.0%)	Sea-surface temperature (18.5%)	Sea-bottom salinity (17.6%)	Sea-bottom salinity (18.1%)
Sea-bottom salinity (9.5%)	Sea-bottom salinity (9.3%)	Sea-surface temperature (13.7%)	Sea-surface temperature (14.1%)
Baltic Sea			
*2017*	*2018*	*2019*	*2020*
Sea-bottom temperature (31.6%)	**Net Primary Production (26.2%)**	Sea-bottom temperature (28.9%)	Sea-bottom temperature (34.1%)
Sea-surface temperature (30.8%)	Sea-bottom temperature (25.9%)	**Net Primary Production (25.5%)**	Sea-surface temperature (29.8%)
**Net Primary Production (15.9%)**	Sea-surface temperature (17.2%)	Sea-surface temperature (22.1%)	**Net Primary Production (24.7%)**
Sea-surface salinity (8.5%)	Sea-surface salinity (14.4%)	**Sea-bottom dissolved oxygen (11.3%)**	**Sea-bottom dissolved oxygen (6.0%)**
Sea-bottom salinity (6.7%)	Sea-bottom salinity (12.2%)	Sea-surface salinity (7.4%)	Sea-surface salinity (4.0%)
**Sea-bottom dissolved oxygen (6.4%)**	**Sea-bottom dissolved oxygen (4.1%)**	Sea-bottom salinity (4.7%)	Sea-bottom salinity (1.6%)
North Sea			
*2017*	*2018*	*2019*	*2020*
**Sea-bottom dissolved oxygen (83.3%)**	**Sea-bottom dissolved oxygen (62.6%)**	**Sea-bottom dissolved oxygen (63.9%)**	**Sea-bottom dissolved oxygen (68.6%)**
**Net Primary Production (16.7%)**	**Net Primary Production (37.4%)**	**Net Primary Production (36.1%)**	**Net Primary Production (31.4%)**
Bay of Biscay			
2017	2018	2019	2020
Sea-bottom dissolved oxygen (71.2%)	Net Primary Production (51.2%)	Net Primary Production (50.6%)	Sea-bottom dissolved oxygen (91.6%)
Sea-bottom temperature (26.0%)	Sea-bottom dissolved oxygen (47.2%)	Sea-bottom dissolved oxygen (49.4%)	Net Primary Production (8.4%)
Net Primary Production (2.9%)	Sea-bottom temperature (1.7%)		
Black Sea			
2017	2018	2019	2020
Sea-bottom dissolved oxygen (42.3%)	Net Primary Production (29.0%)	Net Primary Production (40.9%)	Sea-bottom dissolved oxygen (57.8%)
Net Primary Production (40.2%)	Sea-bottom temperature (28.0%)	Sea-bottom dissolved oxygen (35.6%)	Sea-bottom temperature (41.5%)
Sea-bottom temperature (17.5%)	Sea-bottom dissolved oxygen (26.7%)	Sea-bottom temperature (23.5%)	
	Sea-surface temperature (16.3%)		
Levantine Sea			
2017	2018	2019	2020
Sea-bottom dissolved oxygen (55.2%)	Sea-bottom dissolved oxygen (55.3%)	Sea-surface temperature (46.4%)	Sea-surface temperature (49.1%)
Sea-bottom temperature (44.8%)	Sea-bottom temperature (44.7%)	Net Primary Production (29.4%)	Net Primary Production (28.9%)
		Sea-bottom temperature (24.2%)	Sea-bottom temperature (22.0%)
Western Mediterranean Sea			
2017	2018	2019	2020
Net Primary Production (33.0%)	Sea-surface temperature (38.7%)	Sea-bottom temperature (30.9%)	Sea-bottom temperature (34.5%)
Sea-bottom temperature (26.9%)	Sea-bottom temperature (32.3%)	Sea-surface temperature (24.2%)	Sea-surface temperature (27.1%)
Sea-surface temperature (23.8%)	Sea-bottom dissolved oxygen (16.9%)	Net Primary Production (22.7%)	Sea-bottom dissolved oxygen (20.8%)
Sea-bottom dissolved oxygen (16.3%)	Net Primary Production (12.1%)	Sea-bottom dissolved oxygen (22.3%)	Net Primary Production (17.6%)

The Adriatic and Aegean seas and the Baltic and North Sea are grouped because of their habitat similarities. Years in italics indicate the years of habitat similarity, and bold-highlighted years indicate habitat dissimilarity. The parameters mainly responsible for habitat similarity/dissimilarity are highlighted in bold.

Habitat similarity between the North Sea and the Baltic Sea over the years mainly depended on the net primary production and sea-bottom dissolved oxygen distributions. These two were the only shared parameters between the regions that contributed to the PCA loadings. Although the parameter contribution ranking over the years in the Baltic Sea was variable, the similarity was overall good because of similar net primary production and sea-bottom dissolved oxygen distributions.

The parameter contribution rankings over the years across the other regions were variable. An abrupt change occurred in the Levantine Sea, where sea-bottom dissolved oxygen and temperature weights decreased from 2018 to 2019 and the net primary production and sea-surface temperature weights increased contextually. These variations likely corresponded to a dissolved oxygen reduction (and variance reduction) in the region caused by the peculiar Levantine Sea thermohaline flux^105^. This flux is indeed characterised by dissolved oxygen being inversely correlated with sea-surface temperature and directly correlated with deep-layer temperature increase^106^.

## Usage Notes

ESRI-GRID ASCII files can be visualised with GIS software, e.g., QGIS^29^, or ArcGIS^30^, by dragging and dropping files to the software interface.

## Data Availability

Software to transform text files into ESRI-GRID ASC files is openly available on the GitHub^40^. Software to calculate Habitat Representativeness Score and PCA loadings is also openly available on the GitHub^92^ and through a Web interface in the D4Science e-Infrastructure (RPrototypingLab VRE)^93^. R scripts to calculate cross-correlation and parameter statistics are available on our public Figshare repository^54^, in the “Script and Related Software” dataset.

## References

[1] EU Commission. Achieve Good Environmental Status. *EU Commission Web site* https://ec.europa.eu/environment/marine/good-environmental-status/index_en.htm (2008).

[2] Olenin, S. *et al*. Marine strategy framework directive. *Task Group* **2** (2010).

[3] Long R (2011). The marine strategy framework directive: a new european approach to the regulation of the marine environment, marine natural resources and marine ecological services. Journal of Energy & Natural Resources Law.

[4] Borja A (2013). Good environmental status of marine ecosystems: what is it and how do we know when we have attained it?. Marine Pollution Bulletin.

[5] Froese R, Demirel N, Coro G, Kleisner KM (2017). & Winker, H. Estimating fisheries reference points from catch and resilience. Fish and Fisheries.

[6] Coro, G. Open science and artificial intelligence supporting blue growth. *Environmental Engineering & Management Journal (EEMJ)* **19** (2020).

[7] JRC. EU data collection Web site https://datacollection.jrc.ec.europa.eu/ - Accessed June 2022 (2021).

[8] EcoScope Consortium. The EcoScope EU project Web site https://ecoscopium.eu/ - Accessed June 2022 (2021).

[9] Pikitch EK (2004). Ecosystem-based fishery management. Science.

[10] McLeod, K. L. & Leslie, H. M. Why ecosystem-based management. *Ecosystem-based management for the oceans* 3–12 (2009).

[11] Coro G, Magliozzi C, Ellenbroek A, Pagano P (2015). Improving data quality to build a robust distribution model for architeuthis dux. Ecological modelling.

[12] Coro G, Ellenbroek A, Pagano P (2021). An open science approach to infer fishing activity pressure on stocks and biodiversity from vessel tracking data. Ecological Informatics.

[13] Elith J, Leathwick JR (2009). Species distribution models: ecological explanation and prediction across space and time. Annual Review of Ecology, Evolution and Systematics.

[14] Coro, G., Bove, P. & Ellenbroek, A. Habitat distribution change of commercial species in the adriatic sea during the covid-19 pandemic. *Ecological Informatics* 101675 (2022). 10.1016/j.ecoinf.2022.101675 PMC9123804 35615467

[15] Stanton JC, Pearson RG, Horning N, Ersts P, Reşit Akçakaya H (2012). Combining static and dynamic variables in species distribution models under climate change. Methods in Ecology and Evolution.

[16] Coro G, Magliozzi C, Ellenbroek A, Kaschner K, Pagano P (2016). Automatic classification of climate change effects on marine species distributions in 2050 using the aquamaps model. Environmental and ecological statistics.

[17] Coro G, Pagano P, Ellenbroek A (2020). Detecting patterns of climate change in long-term forecasts of marine environmental parameters. International Journal of Digital Earth.

[18] Wayte SE (2013). Management implications of including a climate-induced recruitment shift in the stock assessment for jackass morwong (nemadactylus macropterus) in south-eastern australia. Fisheries Research.

[19] Tanaka, K. R. Integrating environmental information into stock assessment models for fisheries management. *Predicting Future Oceans* 193–206 (2019).

[20] Szuwalski CS (2016). & Hollowed, A. B. Climate change and non-stationary population processes in fisheries management. ICES Journal of Marine Science.

[21] Bevilacqua AHV, Carvalho AR, Angelini R, Christensen V (2016). More than anecdotes: fishers’ ecological knowledge can fill gaps for ecosystem modeling. PLoS One.

[22] Heymans JJ (2016). Best practice in ecopath with ecosim food-web models for ecosystem-based management. Ecological Modelling.

[23] Piroddi C (2017). Historical changes of the mediterranean sea ecosystem: modelling the role and impact of primary productivity and fisheries changes over time. Scientific reports.

[24] Campana, E. F., Ciappi, E. & Coro, G. The role of technology and digital innovation in sustainability and decarbonization of the blue economy. *Bulletin of Geophysics and Oceanography* 123 (2021).

[25] Van Vuuren DP (2011). The representative concentration pathways: an overview. Climatic change.

[26] Intergovernmental Panel on Climate Change https://www.academia.edu/download/60673993/climate_change_emission_Special_scenarios20190922-59363-1j1i98f.pdf - Accessed October 2022. IPCC Special Report (2000).

[27] Scarcella, G. *et al*. The potential effects of covid-19 lockdown and the following restrictions on the status of eight target stocks in the adriatic sea. *Frontiers in Marine Science* 1963 (2022).

[28] Wikipedia. ESRI-GRID formats description. *Wikipedia* https://en.wikipedia.org/wiki/Esri_grid (2022).

[29] QGIS. Qgis software version 3.20.0. *QGIS Web site* https://www.qgis.org/en/site/ (2022).

[30] ESRI. Arcgis software version 10.7. *ArcGIS Web site* https://www.esri.com/it-it/arcgis/products/arcgis-desktop/overview (2022).

[31] American Museum of Natural History. Maxent software for modelling species distributions. *AMNH Web site* https://biodiversityinformatics.amnh.org/open_source/maxent/ (2022).

[32] Christensen V (2005). Ecopath with ecosim: a user’s guide. Fisheries Centre, University of British Columbia, Vancouver.

[33] Coll, M., Bundy, A. & Shannon, L. J. Ecosystem modelling using the ecopath with ecosim approach. In *Computers in fisheries research*, 225–291 (Springer, 2009).

[34] Colléter M (2015). Global overview of the applications of the ecopath with ecosim modeling approach using the ecobase models repository. Ecological Modelling.

[35] VanDerWal J, Falconi L, Januchowski S, Shoo L, Storlie C (2014). Sdmtools: Species distribution modelling tools: Tools for processing data associated with species distribution modelling exercises. R package version.

[36] US National Institutes of Health. ImageJ software for image analysis with Java and the Terrain Cartography plugin for reading ASC files. *ImageJ Web site* https://imagej.nih.gov/ij/index.html (2018).

[37] GDAL. Translator library for raster and vector geospatial data, version 3.5.0. *GDAL Web site* https://gdal.org/ (2022).

[38] Claus S (2014). Marine regions: towards a global standard for georeferenced marine names and boundaries. Marine Geodesy.

[39] VLIZ. World marine regions definitions and geospatial data. *Marine Regions Web site* www.marineregions.org (2022).

[40] Coro, G. The ASCFileManagement GitHub repository. *GitHub* https://github.com/cybprojects65/ASCFileManagement (2022).

[41] Ready J (2010). Predicting the distributions of marine organisms at the global scale. Ecological Modelling.

[42] Selig ER (2014). Global priorities for marine biodiversity conservation. PloS one.

[43] O’hara CC, Afflerbach JC, Scarborough C, Kaschner K, Halpern BS (2017). Aligning marine species range data to better serve science and conservation. PLoS One.

[44] Scarponi P, Coro G, Pagano P (2018). A collection of aquamaps native layers in netcdf format. Data in brief.

[45] CMEMS. Copernicus Marine Service ocean products data. *Copernicus Marine Service Web site* https://marine.copernicus.eu/ (2022).

[46] E.U. Copernicus Marine Service Information (2021). Global Ocean 1/12° Physics Analysis and Forecast updated Daily. Copernicus Marine Service Web site.

[47] E.U. Copernicus Marine Service Information (2021). Global Ocean Biogeochemistry Analysis and Forecast. Copernicus Marine Service Web site.

[48] Hijmans, R. J. *et al*. Terra: Spatial data analysis. *R Spatial Data Science Web site* https://rspatial.org/terra/ (2022).

[49] MacLeod CD (2010). Habitat representativeness score (hrs): a novel concept for objectively assessing the suitability of survey coverage for modelling the distribution of marine species. Journal of the Marine Biological Association of the United Kingdom.

[50] Abdi H, Williams LJ (2010). Principal component analysis. Wiley interdisciplinary reviews: computational statistics.

[51] Coro G, Pagano P, Ellenbroek A (2013). Combining simulated expert knowledge with neural networks to produce ecological niche models for latimeria chalumnae. Ecological modelling.

[52] Coro, G., Pagano, P. & Ellenbroek, A. Automatic procedures to assist in manual review of marine species distribution maps. In *International Conference on Adaptive and Natural Computing Algorithms*, 346–355 (Springer, 2013).

[53] Magliozzi C, Coro G, Grabowski RC, Packman AI, Krause S (2019). A multiscale statistical method to identify potential areas of hyporheic exchange for river restoration planning. Environmental Modelling & Software.

[54] Coro G, Bove P (2022). FigShare.

[55] Coro, G. Means, standard deviations, geometric means, and log-normal standard deviation of the data produced for the present publication. *D4Science distributed storage system* https://data.d4science.net/foLS (2022).

[56] Mann ME, Bradley RS, Hughes MK (1998). Global-scale temperature patterns and climate forcing over the past six centuries. Nature.

[57] Biskaborn BK (2019). Permafrost is warming at a global scale. Nature communications.

[58] Nemani RR (2003). Climate-driven increases in global terrestrial net primary production from 1982 to 1999. science.

[59] Huang Y, Zhang W, Sun W, Zheng X (2007). Net primary production of chinese croplands from 1950 to 1999. Ecological Applications.

[60] Sunlu U, Aksu M, Buyukisik B, Sunlu FS (2008). Spatio-temporal variations of organic carbon and chlorophyll degradation products in the surficial sediments of izmir bay (aegean sea/turkey). Environmental monitoring and assessment.

[61] Kubryakov A, Mikaelyan A, Stanichny S, Kubryakova E (2020). Seasonal stages of chlorophyll-a vertical distribution and its relation to the light conditions in the black sea from bio-argo measurements. Journal of Geophysical Research: Oceans.

[62] Gamo T (1999). Global warming may have slowed down the deep conveyor belt of a marginal sea of the northwestern pacific: Japan sea. Geophysical Research Letters.

[63] Mahaffey C, Palmer M, Greenwood N, Sharples J (2020). Impacts of climate change on dissolved oxygen concentration relevant to the coastal and marine environment around the uk. MCCIP Science Review.

[64] Zhang W, Dunne JP, Wu H, Zhou F, Huang D (2022). Using timescales of deficit and residence to evaluate near-bottom dissolved oxygen variation in coastal seas. Journal of Geophysical Research: Biogeosciences.

[65] Helm, K. P., Bindoff, N. L. & Church, J. A. Changes in the global hydrological-cycle inferred from ocean salinity. *Geophysical Research Letters* **37** (2010).

[66] Ren, L., Speer, K. & Chassignet, E. P. The mixed layer salinity budget and sea ice in the southern ocean. *Journal of Geophysical Research: Oceans* **116** (2011).

[67] Mahmuduzzaman M (2014). Causes of salinity intrusion in coastal belt of bangladesh. International Journal of Plant Research.

[68] Podymov O, Zatsepin A, Ocherednik V (2021). Increase of temperature and salinity in the active layer of the north-eastern black sea from 2010 to 2020. Physical Oceanography.

[69] Mizyuk, A. & Puzina, O. Sea ice modeling in the sea of azov for a study of long-term variability. In *IOP Conference Series: Earth and Environmental Science*, **vol. 386**, 012023 (IOP Publishing, 2019).

[70] Pärn O, Friedland R, Rjazin J, Stips A (2022). Regime shift in sea-ice characteristics and impact on the spring bloom in the baltic sea. Oceanologia.

[71] Lundesgaard Ø, Sundfjord A, Renner AH (2021). Drivers of interannual sea ice concentration variability in the atlantic water inflow region north of svalbard. Journal of Geophysical Research: Oceans.

[72] Schwegmann, S. & Holfort, J. Regional distributed trends of sea ice volume in the baltic sea for the 30-year period 1982 to 2019. *Meteorologische Zeitschrift* 33–43 (2021).

[73] Simon, S. Interpretation of the correlation coefficient. *PMean Web site* http://www.pmean.com/definitions/correlation.htm (2020).

[74] Yacobi Y (1995). Chlorophyll distribution throughout the southeastern mediterranean in relation to the physical structure of the water mass. Journal of Marine Systems.

[75] Kucuksezgin F, Balci A, Kontas A, Altay O (1995). Distribution of nutrients and chlorophyll-a in the aegean sea. Oceanologica Acta.

[76] Villate F, Aravena G, Iriarte A, Uriarte I (2008). Axial variability in the relationship of chlorophyll a with climatic factors and the north atlantic oscillation in a basque coast estuary, bay of biscay (1997–2006). Journal of Plankton Research.

[77] Iriarte A (2010). Dissolved oxygen in contrasting estuaries of the bay of biscay: effects of temperature, river discharge and chlorophyll a. Marine Ecology Progress Series.

[78] Stanev EV (2005). Black sea dynamics. Oceanography.

[79] Tsimplis MN, Rixen M (2002). Sea level in the mediterranean sea: The contribution of temperature and salinity changes. Geophysical research letters.

[80] Schneider, A., Wallace, D. W. & Körtzinger, A. Alkalinity of the mediterranean sea. *Geophysical Research Letters* **34** (2007).

[81] Sara G, Porporato EM, Mangano MC, Mieszkowska N (2018). Multiple stressors facilitate the spread of a non-indigenous bivalve in the mediterranean sea. Journal of Biogeography.

[82] Soto-Navarro J (2020). Evolution of mediterranean sea water properties under climate change scenarios in the med-cordex ensemble. Climate Dynamics.

[83] Dietze H, Löptien U (2021). Retracing hypoxia in eckernförde bight (baltic sea). Biogeosciences.

[84] Ulses C (2021). Oxygen budget of the north-western mediterranean deep-convection region. Biogeosciences.

[85] Jaskulak M, Sotomski M, Michalska M, Marks R, Zorena K (2022). The effects of wastewater treatment plant failure on the gulf of gdansk (southern baltic sea). International Journal of Environmental Research and Public Health.

[86] Mihanović H (2021). Observation, preconditioning and recurrence of exceptionally high salinities in the adriatic sea. Frontiers in Marine Science.

[87] De Leo F, Besio G, Mentaschi L (2021). Trends and variability of ocean waves under rcp8. 5 emission scenario in the mediterranean sea. Ocean Dynamics.

[88] Omar AM (2019). Trends of ocean acidification and pco2 in the northern north sea, 2003–2015. Journal of Geophysical Research: Biogeosciences.

[89] Kröncke, I. *et al*. Comparison of biological and ecological long-term trends related to northern hemisphere climate in different marine ecosystems. *Nature Conservation* (2019).

[90] Bonnet D (2007). Comparative seasonal dynamics of centropages typicus at seven coastal monitoring stations in the north sea, english channel and bay of biscay. Progress in oceanography.

[91] Borja Á (2011). Implementation of the european marine strategy framework directive: A methodological approach for the assessment of environmental status, from the basque country (bay of biscay). Marine Pollution Bulletin.

[92] Coro, G. An open-source re-implementation of the habitat representativeness score. *GitHub* https://github.com/cybprojects65/HabitatRepresentativenessScore (2022).

[93] Coro, G. An OGC-WPS compliant interface to calculate Habitat Representativeness Score. *D4Science RPrototypingLab VRE* https://services.d4science.org/group/rprototypinglab/data-miner?OperatorId = org.gcube.dataanalysis.wps.statisticalmanager.synchserver.mappedclasses.transducerers.HABITAT_REPRESENTATIVENESS_SCORE (2022).

[94] Assante M (2019). Enacting open science by d4science. Future Generation Computer Systems.

[95] Assante M (2019). The gcube system: delivering virtual research environments as-a-service. Future Generation Computer Systems.

[96] Assante, M. *et al*. Virtual research environments co-creation: The d4science experience. *Concurrency and Computation: Practice and Experience* e6925 (2022).

[97] Coro G, Candela L, Pagano P, Italiano A, Liccardo L (2015). Parallelizing the execution of native data mining algorithms for computational biology. Concurrency and Computation: Practice and Experience.

[98] Coro G, Panichi G, Scarponi P, Pagano P (2017). Cloud computing in a distributed e-infrastructure using the web processing service standard. Concurrency and Computation: Practice and Experience.

[99] Gačić, M., Borzelli, G. E., Civitarese, G., Cardin, V. & Yari, S. Can internal processes sustain reversals of the ocean upper circulation? the ionian sea example. *Geophysical research letters* **37** (2010).

[100] Grilli F (2020). Seasonal and interannual trends of oceanographic parameters over 40 years in the northern adriatic sea in relation to nutrient loadings using the emodnet chemistry data portal. Water.

[101] Cozzi S (2020). Climatic and anthropogenic impacts on environmental conditions and phytoplankton community in the gulf of trieste (northern adriatic sea). Water.

[102] Ducrotoy J-P, Elliott M (2008). The science and management of the north sea and the baltic sea: Natural history, present threats and future challenges. Marine pollution bulletin.

[103] Dupont N, Aksnes DL (2013). Centennial changes in water clarity of the baltic sea and the north sea. Estuarine, Coastal and Shelf Science.

[104] Dippner JW, Möller C, Hänninen J (2012). Regime shifts in north sea and baltic sea: a comparison. Journal of Marine Systems.

[105] Sisma-Ventura, G. *et al*. Post-eastern mediterranean transient oxygen decline in the deep waters of the southeast mediterranean sea supports weakening of ventilation rates. *Frontiers in Marine Science* 1202 (2021).

[106] Mavropoulou A-M, Vervatis V, Sofianos S (2020). Dissolved oxygen variability in the mediterranean sea. Journal of Marine Systems.

[107] Tyberghein L (2012). Bio-oracle: a global environmental dataset for marine species distribution modelling. Global ecology and biogeography.

[108] Assis J (2018). Bio-oracle v2. 0: Extending marine data layers for bioclimatic modelling. Global Ecology and Biogeography.

[109] Coro G, Bove P (2022). A high-resolution global-scale model for covid-19 infection rate. ACM Transactions on Spatial Algorithms and Systems (TSAS).

[110] Inness A (2019). The cams reanalysis of atmospheric composition. Atmospheric Chemistry and Physics.

[111] Karger DN, Schmatz DR, Dettling G, Zimmermann NE (2020). High-resolution monthly precipitation and temperature time series from 2006 to 2100. Scientific data.

[112] Kesner-Reyes, K. *et al*. AquaMaps Environmental Dataset: Half-Degree Cells Authority File (HCAF ver. 7, 10/2019). *AquaMaps Web site* https://www.aquamaps.org/main/envt_data.php (2019).

[113] Kesner-Reyes, K. *et al*. AquaMaps Environmental Dataset: Half-Degree Cells Authority File (HCAF ver. 6, 08/2016). *AquaMaps Web site* https://www.aquamaps.org/main/envt_data.php (2016).

[114] NASA-NEX. NASA Earth Exchange data. *NASA-NEX Web site* https://www.nasa.gov/nex/data - data were publicly accessible up to 2020 (2020).

[115] Coro G (2020). A global-scale ecological niche model to predict sars-cov-2 coronavirus infection rate. Ecological modelling.

[116] CAMS. Global inversion-optimised greenhouse gas fluxes and concentrations. *Copernicus Atmosphere Web site* https://ads.atmosphere.copernicus.eu/cdsapp#/dataset/cams-global-greenhouse-gas-inversion?tab=doc (2020).

[117] NOAA. ETOPO2 Topography and Bathymetry. *NOAA Web site* https://sos.noaa.gov/catalog/datasets/etopo2-topography-and-bathymetry-natural-colors/ (2010).

[118] Coro G, Trumpy E (2020). Predicting geographical suitability of geothermal power plants. Journal of Cleaner Production.

[119] NOAA. World Vector Shorelines. *NOAA Web site* https://shoreline.noaa.gov/data/datasheets/wvs.html (2019).

[120] Tozer B (2019). Global bathymetry and topography at 15 arc sec: Srtm15+. Earth and Space Science.

[121] Ramesh R (2015). Land–ocean interactions in the coastal zone: Past, present & future. Anthropocene.

[122] Spalding, M. *et al*. *World atlas of coral reefs* (Univ of California Press, 2001).

[123] Laske G (1997). A global digital map of sediment thickness. Eos Trans. AGU.

[124] Davies JH (2013). Global map of solid earth surface heat flow. Geochemistry, Geophysics, Geosystems.

[125] Rybach, L. & Muffler, L. J. P. Geothermal systems: principles and case histories. *Chichester, Sussex, England and New York, Wiley-Interscience, 1981. 371 p*. (1981).

[126] Glassley, W. E. Geology and hydrology of geothermal energy. *Power Stations Using Locally Available Energy Sources: A Volume in the Encyclopedia of Sustainability Science and Technology Series, Second Edition* 23–34 (2018).

[127] Barbier E (2002). Geothermal energy technology and current status: an overview. Renewable and sustainable energy reviews.

[128] Engdahl ER, van der Hilst R, Buland R (1998). Global teleseismic earthquake relocation with improved travel times and procedures for depth determination. Bulletin of the Seismological Society of America.

[129] Engdahl, E. R. Global seismicity: 1900–1999. *International handbook of earthquake and engineering seismology* 665–690 (2002).

[130] Richts, A., Struckmeier, W. F. & Zaepke, M. WHYMAP and the groundwater resources map of the world 1: 25,000,000. In *Sustaining groundwater resources*, 159–173 (Springer, 2011).

[131] Warszawski, L. *et al*. Center for international earth science information network—ciesin—columbia university.(2016). gridded population of the world, version 4 (gpwv4): Population density. palisades. ny: Nasa socioeconomic data and applications center (sedac). *Atlas of Environmental Risks Facing China Under Climate Change* 228, 10.7927/h4np22dq (2017).

